# Phytotoxicity of nanoparticles—problems with bioassay choosing and sample preparation

**DOI:** 10.1007/s11356-014-2865-0

**Published:** 2014-04-23

**Authors:** Izabela Jośko, Patryk Oleszczuk

**Affiliations:** Department of Environmental Chemistry, Maria Curie-Skłodowska University, 3 Maria Curie-Skłodowska Square, 20-031 Lublin, Poland

**Keywords:** Phytotoxicity, Engineered nanoparticles, Methods, *Lepidium sativum*

## Abstract

For a full estimation of the risk related with the presence of engineered nanoparticles (ENPs) in the environment, the use of the current ecotoxicological methods may prove insufficient. In the study presented herein, various methods of assessment of ecotoxicity were applied to compare the phytotoxicity of three ENPs: nano-ZnO, nano-TiO_2_ and nano-Ni. The toxicity was assayed both for aqueous solutions of the ENPs (the germination/elongation test and Phytotestkit F^TM^) and for ENPs added to soil (Phytotoxkit F^TM^ and modified Phytotoxkit F^TM^). *Lepidium sativum* was used as a test plant. The scope of the study also included the assessment of the effect of the method of ENP application to the soil (as powder and aqueous suspension) on their phytotoxicity. In the course of the study, no effect of the studied ENPs and their bulk counterparts on the germination of seeds was observed. The root growth inhibition of *L. sativum* depended on the kind of test applied. The trend between concentration of ENPs and effect depended on the method used and kind of ENPs. For most nanoparticles (despite of the method used), the differences in phytotoxicity between nano and bulk particles were observed. Depending on the kind of ENPs, their phytotoxicity differs between water and soil. ZnO (nano and bulk) and nano-Ni were more toxic in soil than in water. For TiO_2_ and bulk-Ni, reverse trend was observed. A different method of ENP application to soil differently affects the phytotoxicity.

**Electronic supplementary material:**

The online version of this article (doi:10.1007/s11356-014-2865-0) contains supplementary material, which is available to authorized users.

## Introduction

The increasing production of engineered nanoparticles (ENPs) entails the risk of their release to the environment (Gottschalk and Nowack 2011), creating a threat to living organisms (Bhatt and Tripathi 2011). Inorganic ENPs, even though they have the same chemical composition as their bulk counterparts, differ from them considerably in their properties—specific surface area, reactivity and tendency towards aggregation (Nowack et al. 2012). This denotes that ENPs should be treated as a “new” group of contaminants, which involves also the necessity of developing new methods for the estimation of their ecotoxicological properties. Nano-ZnO and nano-TiO_2_ belong to the group of ENPs, which due to their potentially extensive use, may constitute a threat to the environment (Piccinno et al. 2012). Those ENPs are components of paints, suntan lotions, dyestuffs or photovoltaic cells. The EU requires that by the year 2018, all chemical compounds whose production exceeds 1 metric ton per year should have ecotoxicological characterisation (European Parliament and European Council 2006). The scale of production of nano-ZnO and nano-TiO_2_ in Europe is estimated at 55 and 550 metric ton per year, respectively, which confirms the necessity of their deep ecotoxicological evaluation (Piccinno et al. 2012). Apart from the popular nano-ZnO and nano-TiO_2_, attention is due also to other ENPs, e.g. nano-Ni. Nano-Ni is more and more frequently used in industrial production (catalyzer, battery electrode, electrochromic films, sensors magnetic materials and diesel–fuel additive) and, like the ENPs mentioned earlier, can constitute a threat to the environment (Rao and Sunandana 2008). As opposed to nano-ZnO and TiO_2_, ecotoxicological studies concerning nano-Ni are relatively scarce.

Plants constitute a significant link in ecotoxicological studies, due to their ecological and economic importance. Therefore, assessment of the potential phytotoxicity of ENPs is particularly crucial. In the literature, one can find numerous studies concerning the phytotoxicity of ENPs (El-Temsah and Joner 2012; Jośko and Oleszczuk 2013a, b; Lee et al. 2010a; Lin and Xing 2007). Two research directions are mainly observed: estimation of the phytotoxicity of aqueous solutions of ENPs (Lin and Xing 2007, 2008) and, in recent years, also of the toxicity of ENPs in soils (Dimpka et al. 2012; Du et al. 2011; Jośko and Oleszczuk 2013a), less frequently in other matrices (Jośko and Oleszczuk 2013b). Although studies of the toxicity of ENPs in soil appear to be more justified than hydroponic cultures, they are also more complicated. The toxicity of ENPs in soils can be additionally affected by the soil properties and by the various components that appear in soils (Jośko and Oleszczuk 2013a; Lv et al. 2012). Unfortunately, divergent results are frequently obtained in studies concerned with the evaluation of toxicity of ENPs in aqueous solutions and in soils (El-Temsah and Joner 2012). The disparities observed in phytotoxicity thresholds among the individual studies may be related to differences in particle size, preparation methods or test designs (Lee et al. 2010b). Up till now, no study has been performed that would present comparative results for various tests of phytotoxicity.

Apart from the comparison of different methods, another significant aspect of the methodological approach to the estimation of toxicity of ENPs is the technique of their application to the soil. This problem was addressed by Hund-Rinke et al. (2012), as well as by Waalewijn-Kool et al. (2012). However, the issue still requires additional detail studies. The technique of application of ENPs (especially in the form of powder or aqueous solutions) can affect the distribution of ENPs in the soil, which may then be reflected in the level of the toxic effects. Depending on the source of pollution, ENPs may undergo translocation to the environment in a solid form (e.g. at the sites of their production) or as an aqueous solution (e.g. ENPs washed down with rainfall).

The objective of the study was to compare various widespread used methods of estimation of the phytotoxicity of three ENPs—nano-ZnO, nano-TiO_2_ and nano-Ni. The study included also factors that may have an effect on the toxicity of ENPs, such as their concentration. The results of phytotoxicity of the ENPs were compared with the toxicity of their bulk counterparts. In addition, it was studied how various techniques of application of ENPs to soil affect their phytotoxicity.

## Materials and methods

### Materials

Nanoparticles ZnO (nano-ZnO), TiO_2_ (nano-TiO_2_), Ni (nano-Ni) and their bulk counterparts (bulk-ZnO, bulk-TiO_2_ and bulk-Ni) were purchased from Sigma–Aldrich (St. Louis, USA). The primary particle size of nanoparticles and Zeta potential was as follows: nano-ZnO <100 nm and 1.53 mV; nano-TiO_2_ < 21 nm and −7.30 mV; nano-Ni <100 nm and 15.53 mV. Surface area of nano-ZnO, nano-TiO_2_ and nano-Ni was 15–25, 35–65 and 8–12 m^2^/g, respectively (data from Sigma–Aldrich). The size of nanoparticles was determined by transmission electron microscope (JEM-3010 TEM JEOL, Ltd., Tokyo, Japan). The TEM pictures of nanoparticles used in the experiment are presented elsewhere (Jośko and Oleszczuk 2013a, b).

### Sample preparation for phytotoxicity testing

Aqueous solutions of the ENPs (methods M1 and M2) were prepared with re-distilled water, at the following concentrations: 10, 100 and 1,000 mg/L. Immediately prior to their application onto the test plates (Petri dishes and plates for Phytotestkit F^TM^—please see description later), the solutions containing the ENPs were subjected to 30-min sonication in an ultrasonic bath (25 °C, 250 W, 50 Hz). In parallel, an estimation of the toxicity of ENP bulk counterparts was performed for the concentration of 100 mg/L. The control sample constituted the re-distilled water without any content of the ENPs and their bulk counterparts.

In the experiment concerning the toxicity of the solid fraction (methods M3 and M4), the standard Organisation for Economic Co-operation and Development (OECD) soil was used (MicroBioTests Inc., Mariakerke, Belgium). The OECD artificial soil is a widely used substrate in soil toxicity tests. It has been recommended as a medium for ecotoxicological tests, and it is a “reference soil” in the testing of complex solid samples (e.g. wastes or contaminated soils). OECD artificial soil is defined as a mixture of 70 % fine quartz sand (50 % particles, 0.05–0.2 mm), 20 % kaolin clay (kaolinite content preferably above 30 %) and finely ground Sphagnum peat.

The ENPs were added to the soil as powder, in doses of 10, 100 and 1,000 mg/kg. The soil with ENPs was thoroughly mixed with a glass spatula and rolled end over end (Rotax 6.8, VELP—OMC, ENVAG) for 24 h (in the dark at room temperature) at 10 rpm. The mixtures of OECD soil and ENPs were used immediately after mixing. The phytotoxicity of the ENPs was compared with the phytotoxicity of their bulk counterparts for the concentration of 100 mg/kg. The control sample was the soil without any content of ENPs and their bulk counterparts.

Methods M2 and M3 were chosen for the comparison of phytotoxicity of the ENPs in aqueous solutions and in the soil. In both methods, the same kind of test plates was used, filled with either spongy insert (M2) or soil (M3). For comparison of the two methods, the ENPs or their bulk counterparts were added to the plates in the same amount, i.e. 10.8 mg. In method M2, 10.8 mg of ENPs or bulk counterparts was added to 20 mL of re-distilled water (in conformance with the requirements of Phytotestkit F^TM^—please see description below). Next, the solution was subjected to 30-min sonication in an ultrasonic bath (25 °C, 250 W, 50 Hz) and evenly distributed on the spongy insert constituting a component of the test. In method M3, the ENPs or bulk counterparts (10.8 mg) were added to the soil (108.8 g) and rolled end over end (Rotax 6.8, VELP—OMC, ENVAG) for 24 h (in the dark at room temperature) at 10 rpm. Next, the sample was spread on a test plate. The concentration of the ENPs in the soil was 100 mg/kg.

### Phytotoxicity depending on method of ENP application

In the experiment with the effect of method of ENP application, ENPs were applied into the OECD soil as (1) a powder, (2) a water suspension with then soil being dried and tested and (3) a water suspension without soil drying. Final concentration of ENPs in each method corresponded to 100 mg/kg.

In method M1, the required amount of ENPs was weighed and applied directly to the soil, then rolled end over end (Rotax 6.8, VELP—OMC, ENVAG) for 24 h (in the dark at room temperature) at 10 rpm. In method M2, the suspensions of ENPs in redistilled water were sonicated for 30 min (25 °C, 250 W, 50 Hz) and then introduced into the soil. The soil was mixed thoroughly and then dried at a constant temperature (25 °C) until dryness. In method M3, the solution of ENPs after 30 min of sonication (25 °C, 250 W, 50 Hz) was applied uniformly on the surface of the soil placed in the test plate. The concentration of the ENPs was 100 mg/kg of soil. The effect of method of ENP application was estimated using Phytotoxkit F^TM^ (M3).

### Determination of the phytotoxicity

For the estimation of acute phytotoxicity, three methods were applied: the germination/elongation test (OECD 1984), Phytotestkit F^TM^ (MicroBioTests Inc., Belgium), Phytotoxkit F^TM^ (MicroBioTests Inc., Belgium) and Phytotoxkit F^TM^ after our modification. The tests characterise the simplicity in using. In addition, these tests are rapid and cheap, which are especially important in the risk assessment and monitoring of new pollutants in environment. In the first two tests, the phytotoxicity of aqueous solutions was evaluated, while the other two were devoted to the toxicity of the ENPs in the soil. As a test plant, cress (*Lepidium sativum*) was tested, which is frequently used in ecotoxicological studies (Fuentes et al. 2006; Jośko and Oleszczuk 2013a; Walter et al. 2006). Two commonly applied parameters of phytotoxicity were estimated: germination inhibition and root elongation inhibition. All the tests were conducted in accordance with the recommendations of OECD (1984) and with the methodology provided by the producer of the tests (MicroBioTests Inc., Belgium). Regardless of kind of bioassay, the plates of each test were incubated for 3 days at 25 °C in darkness in incubator (Q-CELL, POL-LAB, Warszawa, Poland).

Germination/elongation test (M1) was carried out in accordance (Fig. S1, supporting information) to the OECD procedure (OECD 1984). Seed germination and root elongation test was conducted in glass Petri dishes (90 × 20 mm) with a thin layer of filter paper on the bottom. Each dish contained 5 mL of sample solution (at concentrations of ENPs mentioned above) or 5 mL of redistilled water (control) and 15 seeds.

Phytotestkit F^TM^ (M2) is a new tool for assessment of effluent toxicity proposed by MicroBioTests Inc. (Gent, Belgium). Unique flat and shallow transparent test plates composed of two compartments (Fig. S2, supporting information) are used in this method. The lower plate contains a spongy insert and a thick cellulose filter paper. In the present experiment, 20 mL of ENP solution (at 10, 100 and 1,000 mg/L, which correspond with concentrations used in OECD procedure—M1) was evenly distributed on the thick filter paper surface. Then, a thick filter was covered with a thin filter paper. Ten seeds of *L. sativum* were positioned at equal distance near the middle ridge of test plates on a filter paper. After closing the test plates with their transparent cover by means of the unique click system, the test plates are placed vertically in a holder and incubated.

Phytotoxkit F^TM^ (M3) uses the same test plates as Phytotestkit F^TM^ (Fig. S3, supporting information). The lower compartment of plate contained of tested soil instead of spongy insert saturated to the water holding capacity (for OECD soil is 35 mL). To soil, previously, ENPs were added at appropriate dose to obtain concentrations: 10, 100 and 1,000 mg/kg. On the top of the hydrated soil, a thin filter paper was placed. The next steps were the same like described for Phytotestkit F^TM^ method.

Modified Phytotoxkit F^TM^ (M4) was performed in accordance with test procedures of Phytotoxkit F^TM^ method (Fig. S4, supporting information). However, instead of putting *L. sativum* seeds on the filter paper, they were placed directly on the ENP-contaminated soil.

All bioassays according to the user’s manual were performed in three replicates. At the end of the incubation period, “digital” pictures were taken of the test plates with the germinated plants. Then, the amount of germinated seeds and the length of roots were determined using the Image Tool 3.0 for Windows (UTHSCSA, San Antonio, USA). The percent inhibition of seed germination (SG) and root growth inhibition (RI) was calculated by the formula:

$$ \mathrm{SG}\kern0.5em \mathrm{or}\kern0.5em \mathrm{RI}=\left(\mathbf{\mathcal{A}}-\mathbf{\mathcal{B}}\right)/\mathbf{\mathcal{A}}\times 100, $$

where *A* is the seed germination/ root length in the control sample and *B* is the seed germination/root length in the sample. Statistically significant differences between the results were evaluated on the basis of standard deviation determinations and on the analysis of variance method (Statistica 5.0; StatSoft, Tulsa, OK, USA)

## Results and discussion

In the course of the study, no effect of the studied ENPs and their bulk counterparts on the germination of seeds was observed. In certain cases, germination inhibition was at levels <10 %, and it was not related to the presence of the ENPs. For this reason, that parameter has been omitted in the description and discussion of results. Similarly, El-Temsah and Joner (2012) did not observe any effect of ENPs on the germination of seeds and concluded that root elongation is a more sensitive indicator.

### Influence of ENP concentration on phytotoxicity depending on the test used

**Fig. 1:**
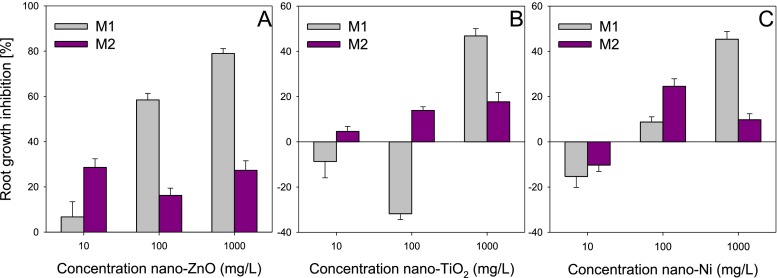
Influence of nano-ZnO (**a**), nano-TiO_2_ (**b**) and nano-Ni (**c**) concentration in water on their phytotoxicity to *Lepidium sativum* depending on test applied (M1—germination/elongation test, M2—Phytotestkit F^TM^). *Error bars* represent standard error (SE, *n* = 3 tests)

The root growth inhibition of *L. sativum* depended on the kind of test applied (Fig. 1). The application of method M1 showed a relationship between the effect observed and the concentration of nano-ZnO. However, when the method M2 was used (Fig. 1a), the relation was not observed. In method M2, the root elongation inhibition of *L. sativum* did not differ significantly between the studied concentrations of nano-ZnO. The results obtained for method M2 were also incomparable in any case with those obtained for method M1. The lack of correlation between the concentration and the toxic effect observed could have been related probably with the test design. In method M1, the solution contained the ENPs was applied directly on a layer of the filter paper, which permitted uniform distribution of the ENPs and direct contact of the seeds with the ENPs (Fig. S1, supporting information). Whereas, in the case of M2, the solution with the ENPs was applied on a thick filter with insert sponge. In this case, the ENPs might penetrate deep into the filter (Fig. S2, supporting information). That limited the direct contact of seeds with the ENPs. Moreover, the seeds were separated from the solution containing the ENPs by the filter, which could have caused accidental contact of the seeds with the ENPs. As it is demonstrated later in the paper, the presence of the filter may reduce the toxicity of ENPs in the method for solid phase (Fig. 3). Only in the case of the lowest concentration, the toxicity of nano-ZnO was higher after the application of method M2 than method M1. For higher concentrations, the root growth inhibition was over twice lower in method M2 than in M1. Depending on the concentration of nano-ZnO, root growth inhibition varied from 6.7 to 78.9 % in method M1, while in method M2, it varied from 16.2 to 28.6 % (Fig. 1a).

Tendencies different from those for nano-ZnO were observed for nano-TiO_2_. A significant correlation between the dose and the effect was observed in method M2 (Fig. 1b), while in method M1, no similar correlation was noted (Fig. 1b). In addition, nano-TiO_2_ assayed with method M1 in concentrations of 10 and 100 mg/L displayed a stimulating effect on root growth, which was not observed in the case of method M2. Nano-TiO_2_ had a toxic effect on *L. sativum*, inhibiting root growth at the levels of 4.6 and 13.9 %. At the highest concentration, both methods clearly indicated toxicity of nano-TiO_2_. As was the case with nano-ZnO, the root growth inhibition determined with method M1 was more than twice as high as that evaluated with method M2, which can be attributed, as in the case of nano-ZnO, to increased contact between the ENPs and the plant roots.

The toxicity of nano-Ni, like that of nano-ZnO, was correlated with concentration only in the method M1, while absence of such a correlation was noted in method M2 where root growth stimulation/inhibition did not exceed 25 % (Fig. 1c). The lowest concentration (10 mg/L) of nano-Ni was the only of the tested doses of all the ENPs in the case of which an absence of significant differences between methods M1 and M2 was observed. In both cases, nano-Ni stimulated the elongation of roots of *L. sativum* (Fig. 1c). Increase of the concentration of nano-Ni to 100 mg/L caused an increase of the toxicity of nano-Ni towards *L. sativum*. However, the toxicity of nano-Ni assayed with method M1 (8.8 %) was threefold lower than in the case of application of method M2 (24.5 %). As for the other ENPs under study, toxicity assayed with method M1 was significantly higher than with method M2 for the highest concentration of nano-Ni (1,000 mg/kg), which could have been the result of different “true” exposure of *L. sativum* to the ENPs.

The differences in the effects demonstrated with methods M1 and M2 (especially for the highest concentration, at which the toxicity of the ENPs determined with method M1 was higher) may result from the fact that in the case of method M1, the seeds (and then the roots) had direct contact with the ENPs, while in method M2, they were separated from the solution with the ENPs by the filter which could limit the contact, especially for larger aggregates. Moreover, as mentioned before, the solution with the ENPs could have penetrated into the insert sponge, thus limiting the contact with the seeds and the roots. It is assumed that the toxic effect caused by ENPs can, on the one hand, result from the release of ions from ENPs (Misra et al. 2012b), but on the other, it can inhibit water transport into the plants due to surface coating of roots by ENPs (Canas et al. 2008; Wu et al. 2012). The results obtained may confirm the cooperation of those causes in the shaping of the toxicity of ENPs. This is indicated by the effects demonstrated in method M2, where the roots of *L. sativum* were separated from the ENPs by the filter. In that case, the roots were exposed only to the ions released from the ENPs that penetrated the barrier of the filter. Similar conclusions were drawn by Van der Vliet et al. (2012) who observed that poorly soluble contaminants cannot pass through a filter, which may cause an overestimation of plant tolerance to them. Although at the lower concentrations, it was method M2 that demonstrated higher toxicity than method M1 (in certain cases), but that could have resulted from the fact that lower aggregation takes place at lower concentrations. This in turn may be responsible for weaker adhesion of the ENPs to the roots (M1) and at the same time for increased release of ions from the ENPs (greater specific surface area and solubility) (M2), and thus for greater toxicity.

**Fig. 2:**
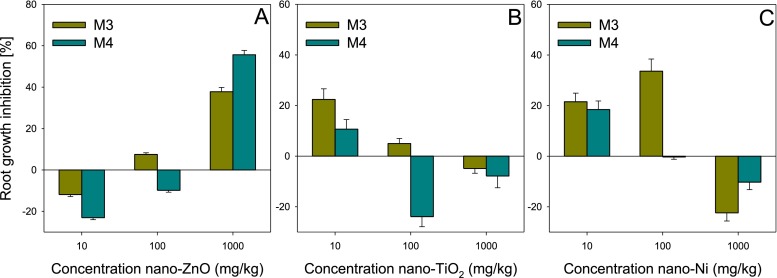
Influence of nano-ZnO (**a**), nano-TiO_2_ (**b**) and nano-Ni (**c**) concentration in soil on their phytotoxicity to *Lepidium sativum* depending on test applied (M3—Phytotoxkit F^TM^, M4—modified Phytotoxkit F^TM^). *Error bars* represent standard error (SE, *n* = 3 tests)

As in the case of the aqueous solutions, also in relation to the solid phase, varied results were observed depending on the method applied (Fig. 2). In both of the methods applied for solid phase (M3 and M4), a dose–effect correlation was observed in the case of nano-ZnO (Fig. 2a). The lower doses of nano-ZnO stimulated the elongation of roots of *L. sativum*, while the higher doses had a toxic effect, which may indicate *hormesis* effect (Calabrese and Baldwin 2003; Manzo et al. 2011). At the highest and the lowest concentrations, in method M4, stimulation (10 mg/kg) or inhibition (1,000 mg/kg) higher by approximately 11–17 % than in method M3 was observed. The difference in those values may result (like in the aqueous solutions) from the fact that in the case of method M4, the seeds were placed directly on the soil, which allowed better access of the ENPs to the plant roots than in method M3, where the seeds were separated from the soil by the filter. Also, Persoone and Wadiha (2010) observed that the elimination of the filter and placement of seeds directly on the soil notably increased the sensitivity of the test Phytotoxkit F^TM^. However, at the concentration of 100 mg/kg, a reverse tendency was observed—method M3 displayed a slight toxicity while according to method M4 nano-ZnO stimulated root elongation. This may be a result of the fact that the ENPs, having direct contact with the plant, can enter into reactions with components of cell walls, forming “tunnels” (Pokhrel and Dubey 2013) that provide passage not only for the ENPs themselves but also for nutrients, stimulating root elongation. That effect may appear at just the concentration of 100 mg/kg, as the aggregation is too great (as for 1,000 mg/kg), which ensures lesser “coating” and pore clogging by the ENPs.

Contrary to nano-ZnO, the effect of nano-TiO_2_ (Fig. 2b) and nano-Ni (Fig. 2c) on the elongation of roots of *L. sativum* did not display a dose–effect correlation. Irrespective of the method applied, the stimulation/inhibition effects did not exceed the levels of 25 % (for nano-TiO_2_) and 35 % (for nano-Ni). It was only at the concentration of 100 mg/kg that differences were observed between the two methods for both ENPs. Like for nano-ZnO, also for nano-TiO_2_ and nano-Ni, method M3 demonstrated higher toxicity compared to method M4 at ENP concentration at the level of 100 mg/kg. As opposed to nano-ZnO, the highest toxic effect was not observed at the highest concentration of nano-TiO_2_ and nano-Ni. The strongest inhibition was noted for the lowest concentration (10 mg/kg), method M3 displaying 12 % higher toxicity for nano-TiO_2_ compared to method M4, while for nano-Ni—irrespective of the method applied—the toxicity was approximately 20 %. In turn, at the highest concentration, nano-TiO_2_ and nano-Ni stimulated root elongation, irrespective of the method applied. In the case of nano-TiO_2_ (contrary to the concentration of 10 mg/kg), no differences were noted between the methods, while for nano-Ni, the stimulation assayed with method M3 was higher by 12 % compared to method M4.

The differences, or their absence, in the levels of toxicity/stimulation between the methods indicate how much important the kind of the ENPs and their concentration is. This is especially observable in the case of nano-TiO_2_ and nano-Ni which at the highest concentration, stimulated root growth in soil, irrespective of the method applied (in the aqueous solution the highest toxicity was observed). Those effects are extremely strange. Even if we assume that ions released from the ENPs at that concentration had a stimulating effect, the question remains concerning the toxicity of those ENPs at the lowest concentration, i.e. 10 mg/kg. Studies conducted so far, especially concerning nano-TiO_2_, confirmed the absence of an effect or a stimulating character of nano-TiO_2_ on the growth of roots of various plants (Larue et al. 2012; Zheng et al. 2005). Whereas, there is a lack of studies on the toxicity of nano-Ni. Perhaps, such a distribution of toxicity of nano-TiO_2_ and nano-Ni is specific for that kind of ENPs, as the effect of nano-ZnO—irrespective of the method applied—displayed the “typical” tendency (dose–effect). It is also worth noting that in both methods, the ENPs were added to the soil in the form of powder, which permitted good homogenisation of the sample (Hund-Rinke et al. 2012). That eliminates the factor of randomness as a cause of the absence of the dose–effect correlation (this is also supported by the low values of standard deviation).

### Effect of ENP comparing to their bulk counterparts 

In the estimation of the toxicity of ENPs, it is of particular importance to determine whether that new group of compounds behaves differently to their already-known bulk counterparts. This is especially important in the context of the various research methods applied. Numerous studies on the toxicity of ENPs show that those compounds are characterised by greater toxicity than their bulk counterparts (Stampoulis et al. 2009; Musante and White 2010; Dimpka et al. 2012). This study includes a comparison of the effect of the particular methods on the differences in toxicity between the nanoparticles and the bulk counterparts.

**Fig. 3:**
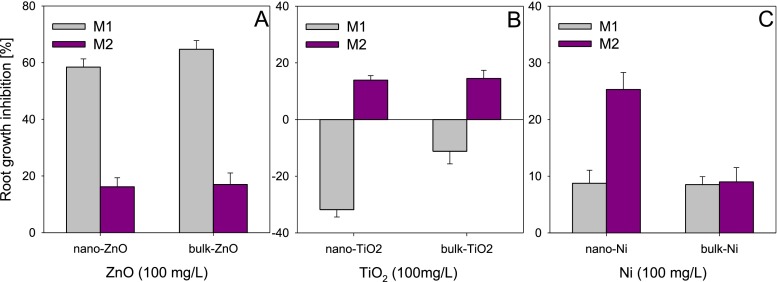
Influence of size (nano or bulk) of ZnO (**a**), TiO_2_ (**b**) and Ni (**c**) on their phytotoxicity to *Lepidium sativum* depending on test applied (M1 and M2). NPs concentration—100 mg/L. *Error bars* represent standard error (SE, *n* = 3 tests)

The aqueous solutions of the ENPs and their bulk counterparts did not differ in the effects within the same method in the case of ZnO (M1 and M2), TiO_2_ (M2) and nano-Ni (M1) (Fig. 3). A study by El-Temsah and Joner (2012) on the phytotoxicity of ENPs also demonstrated the absence of the effect of size, but that could have resulted from the selection of the parameter under estimation (germination inhibition) which turns out to be a little sensitive to the effect of ENPs. Methods M1 and M2 did not reveal differences between nano-ZnO and bulk-ZnO, but the inhibition assayed with method M1 was threefold higher compared to M2 (Fig. 3a). The absence of the effect of size of the ENPs could have been due to similar exposure of *L. sativum* on ions released from the ENPs. As shown in a study by Misra et al. (2012a), the amount of ions released both from ENPs and from their bulk counterparts was similar, which can explain the similar phytotoxicity of nano and bulk particles. Whereas, for TiO_2_ and Ni, the differences (or their absence) between the nano and bulk forms were related to the method applied (Fig. 3b, c). In method M1, both forms of TiO_2_ stimulated root growth, the stimulation by nano-TiO_2_ being twice as high as that caused by bulk-TiO_2_. Schmidt and Vogelsberger (2006) observed an increase in the solubility of nano-TiO_2_ with decrease in the size of the particles, due to which the released ions could exert a stimulating effect on plants (Song et al. 2013). Whereas, the effects assayed with method M2, though toxic, did not differ significantly between the forms of TiO_2_ (the toxic effects were low, <20 %). In turn, for Ni, the determination of toxicity with method M2 alone revealed an effect of size. According to method M1, root growth inhibition by nano-Ni was threefold lower compared to method M2, while for bulk-Ni, no differences were observed between the two methods. This is particularly strange, as it was in method M1 that the plants had direct contact with Ni. Therefore, it should be expected that the toxicity of nano- and bulk-Ni should be higher than that assayed with method M2, as apart from ions released from the ENPs, the particles adhering to the roots could also be responsible for the toxicity.

**Fig. 4:**
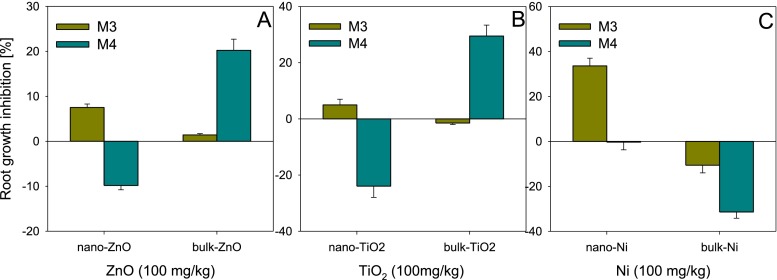
Influence of size (nano or bulk) of ZnO (**a**), TiO_2_ (**b**) and Ni (**c**) on their phytotoxicity to *Lepidium sativum* depending on test applied (M3 and M4). NPs concentration—100 mg/kg. *Error bars* represent standard error (SE, *n* = 3 tests)

The effect of the size of particles on their toxicity in the soil was more varied than in the aqueous solutions (Fig. 4). Only the application of method M3 did not indicate significant differences between the nano and bulk forms for TiO_2_ and ZnO. Method M3 showed low toxicity (<10 %) of both forms of ZnO (Fig. 4a). However, in the soil, even released ions of Zn^2+^ (to which the seeds are exposed only in this case) may not pose a threat to the plants. The ions can be bound by soil components, due to which they are not available for plants, which may support the low level of toxicity of ZnO (Dimpka et al. 2012). On the other hand, notable differences between the nano and bulk forms of ZnO were found in method M4. Nano-ZnO stimulated root elongation of *L. sativum* (9.8 %), while bulk-ZnO caused a 20 % toxic effect (Fig. 4). Direct contact of ZnO with the seeds and roots ensured similar exposure to ions Zn^2+^ as in method M3. Perhaps the cause of the differences in toxicity between nano-ZnO and bulk-ZnO lies in what happens to the ENPs in the soil and in the behaviour of ZnO on root surface of *L. sativum*. In the case of TiO_2_, irrespective of particle size, method M3 showed a slight effect on root elongation (it did not exceed 5 %) (Fig. 4b). Whereas, in method M4, considerable differences in toxicity were found between TiO_2_ forms with different particle size. As in the case of nano-ZnO, the addition of nano-TiO_2_ to the soil strongly stimulated root elongation of *L. sativum* (23.9 %), while the bulk counterparts caused its inhibition (29.5 %). In the case of Ni, both methods revealed strongly varied effects caused by nano-Ni and bulk-Ni (Fig. 4c). In method M3, much greater toxicity was observed for nano-Ni (33.6 %) than for bulk-Ni (which stimulated root elongation). Whereas, in method M4, no effect of nano-Ni on root elongation was observed, while bulk-Ni caused stimulation at the level of 31.4 %. As opposed to both forms of ZnO and TiO_2_, nano- and bulk-Ni displayed an effect of size: the toxic effect of nano-Ni was greater than that of bulk-Ni.

### Phytotoxicity of ENPs in water solution and soil

**Fig. 5:**
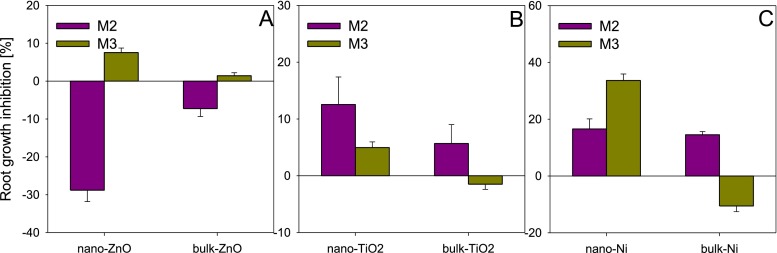
Comparison of the phytotoxicity of ZnO (**a**), TiO_2_ (**b**) and Ni (**c**) in water (Phytotestkit F^TM^—M2) and in soil (Phytotoxkit F^TM^). *Error bars* represent standard error (SE, *n* = 3 tests)

With relation to the kind of phase tested (aqueous solution (M2) or solid phase (M3), the effect of ZnO, TiO_2_ and Ni on the plants was varied (Fig. 5). In the aqueous solution (M2), both forms of ZnO stimulated the growth of *L. sativum*, the stimulation in the presence of nano-ZnO (28.8 %) being several-fold higher than in the case of bulk-ZnO (7.3 %). In the soil samples (M3), on the other hand, an inhibition of root elongation was observed, characterised by a relatively low level (<8 %) (Fig. 5a). An opposite tendency was observed for TiO_2_. Inhibition of root elongation of *L. sativum* was over twice as high for the aqueous solution as for the soil, but the level of toxicity did not exceed 15 % (Fig. 5b). Depending on the matrix tested, root elongation of *L. sativum* in the case of Ni was related to its form (Fig. 5c). Higher toxicity was observed in case of the soil fraction (M3) compared to the aqueous solution (M2) for nano-Ni, while for bulk-Ni, a reverse tendency was observed.

It is commonly known that the toxicity of many contaminants is determined to a significant extent by their interactions with the particular components of the environment. In soil characterised by a greater diversity of various components, the number and kind of probable interactions will be greater than in an aqueous solution. The toxicity of contaminants, including ENPs, depends to a significant degree on their bioavailability (Dimpka et al. 2012; El-Temsah and Joner 2012). Soil has a greater ability to reduce the bioavailability of ENPs than aqueous environments (Pan and Xing 2010). The sorption complex of soils (natural organic matter, clays) can immobilise ENPs, which reduces their availability for plants, and thus also their toxicity (Pan and Xing 2010; Tourinho et al. 2012). Therefore, it should be expected that the toxicity of ENPs in soil will be lower than in water, due to the limitations resulting from reduced bioavailability. Such regularity was observed for both forms of TiO_2_ and bulk-Ni. It should be kept in mind, however, that the values did not exceed the level of 13 %, which makes it difficult to formulate definitive conclusions. Whereas, in this study, lower toxicity of both forms of ZnO and nano-Ni was noted in the aqueous solutions than in the soil. Perhaps, this is related with the test design. In method M2, the nano and bulk particles were applied on the filter, while in method M3, directly to the soil. Soil can contain components that cause an increase in the solubility of ENPs (Lv et al. 2012; Misra et al. 2012b), thanks to which ions could penetrate through the filter. No increase in the solubility of the ENPs was observed in method M3 due to the absence of those components in the spongy insert, which could have caused the differences observed.

### Influence of ENP application on their phytotoxicity

**Fig. 6:**
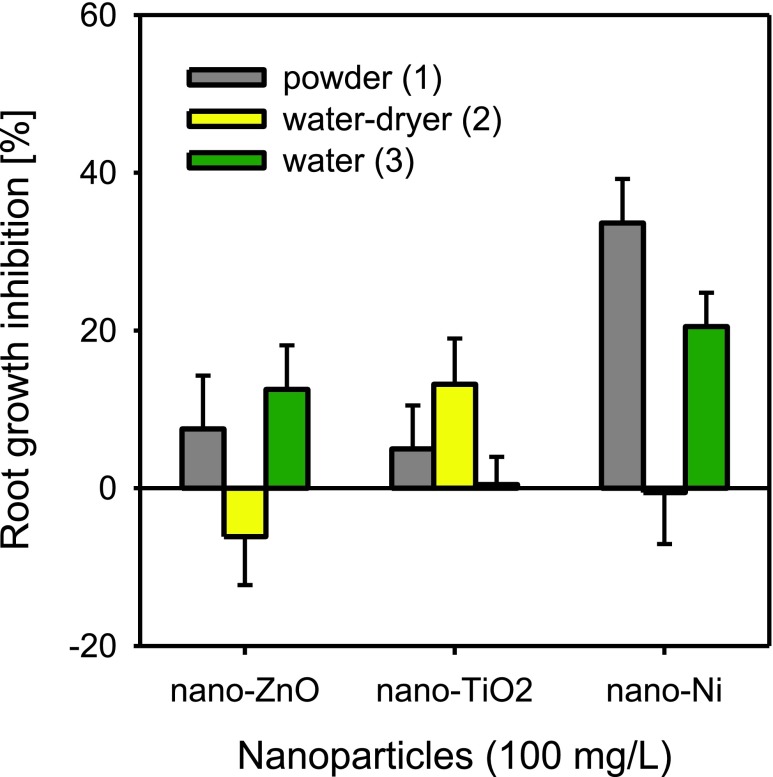
Influence of method of NP application to the soil on their phytotoxicity to *L. sativum. Error bars* represent standard error (SE, *n* = 3 tests)

The method of application of nanoparticles to the soil had a significant effect on the level of toxicity observed in the case of all nanoparticles tested (Fig. 6). The addition of nano-ZnO to soil with methods M1 (ENPs added to soil as powder) and M3 (ENPs added to soil as water suspension without soil drying) displayed similar phytotoxicity at levels of 8 and 13 %, respectively. Application of nano-ZnO with method M2 (water-dryer), on the other hand, had a positive effect on the growth of roots of *L. sativum*, stimulating their growth. A similar tendency was observed in the case of nano-Ni which displayed only a slightly higher toxicity after application with method M1 (34 %) as compared to nano-Ni applied with method M3 (21 %) (Fig. 6). Application of nano-Ni with method M2 produced no effect on root growth and even slightly stimulated root length as it was observed for nano-ZnO. A different situation, compared to nano-ZnO and nano-Ni, was found in the case of nano-TiO_2_. Application of the ENPs with methods M1 and M2 resulted in toxicity at levels of 5 and 13 %, respectively, while nano-TiO_2_ applied with method M3 had no effect on root growth of *L. sativum*. Studies on the toxicity of nanomaterials are not free of many ambiguities concerning also the methodological aspects (Von der Kammer et al. 2012; Waalewijn-Kool et al. 2012). Highly important in this respect is the method of application of ENPs that may determine their toxicity. As demonstrated by the results of the study, depending on the method of application, considerable differences were obtained, attaining as much as 35 %. Studies by Lyon et al. (2006) showed that the method of application had an effect on the antibacterial properties of fullerenes (C_60_). The application of C_60_ in the form of powder had no toxic effect on *Bacillus subtilis*, while applied in water solutions (prepared in various ways), it displayed toxicity towards bacteria expressed in minimal inhibitory concentrations (MIC) at the level of 0.08–0.75 mg/L. Whereas, Waalewijn-Kool et al. (2012) did not observe any significant effect of the method of application of ZnO ENPs on the reproduction of *Folsomia candida.* ENPs are susceptible to aggregation, due to which samples have to be subjected to treatments causing the dispersion of particles so as to enable the verification of toxicity of ENPs and not of their aggregates (Lyon et al. 2006; Von der Kammer et al. 2012). Still, there is a lack of literature data that would permit a clear statement as to the form in which ENPs migrate to or occur in the soil. The method of application of ENPs is also important for another reason, as it demonstrate how the form in which ENPs arrive in soil determines their subsequent behavior and activity towards plants.

## Conclusions

Depending on the method applied, the level of toxicity of the ENPs was strongly varied. The differences in the effects caused by the presence of the ENPs, assayed with different methods, concerned each of the variables under estimation: the kind of ENPs, concentration and the matrix. Comparison of the effects revealed by the particular methods is problematic due to the lack of the dose–effect correlation (especially in the case of nano-TiO_2_, nano-Ni). Also, the use of various techniques of application of the ENPs to the soil diversified the effects caused by the ENPs. The study indicates sometimes extremely different effects of the ENPs on the plants in relation to the method applied. This may lead to erroneous estimations, either underestimating or overestimating the potential threat related with ENPs. The differences in the levels of the effects assayed with the use of different methods confirm the necessity of their validation. This is particularly important in the context of the increasing number of studies on the toxic potential of ENPs. The development of universal ecotoxicological methods for the estimation of toxicity of ENPs would permit the comparison of research results obtained by various research teams. In a more distant perspective, when ENPs become a more common contaminant in the environment, studies on NP based on uniform methods will contribute to the assessment of the scale of the problem in various regions of the world and to the development of legislative regulations.

## Electronic supplementary material

Below is the link to the electronic supplementary material.

ESM 1 (DOCX 1085 kb)

## References

[CR1] Bhatt I, Tripathi BN (2011). Interaction of engineered nanoparticles with various components of the environment and possible strategies for their risk assessment. Chemosphere.

[CR2] Calabrese EJ, Baldwin LA (2003). Hormesis: the dose–response revolution. Annu Rev Pharmacol Toxicol.

[CR3] Canas JE, Long M, Nations S, Vadan R, Dai L, Luo M, Ambikapathi R, Lee EH, Olszyk D (2008). Effects of functionalized and nonfunctionalized single-walled carbon nanotubes on root elongation of select crop species. Environ Toxicol Chem.

[CR4] Dimpka CO, McLean JE, Latta DE, Manangon E, Britt DW, Johnson WP, Boyanov MI, Anderson AJ (2012). CuO and ZnO nanoparticles: phytotoxicity, metal speciation, and induction of oxidative stress in sand-grown wheat. J Nanoparticle Res.

[CR5] Du W, Sun Y, Ji R, Zhu J, Wu J, Guo H (2011). TiO_2_ and ZnO nanoparticles negatively affect wheat growth and soil enzyme activities in agricultural soil. J Environ Monit.

[CR6] El-Temsah YS, Joner EJ (2012). Impact of Fe and Ag nanoparticles on seed germination and differences in bioavailability during exposure in aqueous suspension and soil. Environ Toxicol.

[CR7] European Parliament and European Council (2006) Directive 2006/121/EC. Off J Eur Union 561:L396, 850

[CR8] Fuentes A, Llorens M, Saez J, Aguilar MI, Perez-Marin AB, Ortuno JF, Meseguer VF (2006). Ecotoxicity, phytotoxicity and extractability of heavy metals from different stabilized sewage sludges. Environ Pollut.

[CR9] Gottschalk F, Nowack B (2011). The release of engineered nanomaterials to the environment. J Environ Monitor.

[CR10] Hund-Rinke K, Schlich K, Klawonn T (2012) Influence of application techniques on the ecotoxicological effects of nanomaterials in soil. Environ Sci Eur 24:30 doi:10.1186/2190-4715-24-30

[CR11] Jośko I, Oleszczuk P (2013). Influence of soil type and environmental conditions on the ZnO, TiO2 and Ni nanoparticles phytotoxicity. Chemosphere.

[CR12] Jośko I, Oleszczuk P (2013). The influence of ZnO and TiO_2_ nanoparticles on the toxicity of sewage sludges. Environ Sci Proc Imp.

[CR13] Larue C, Laurette J, Herlin-Boime N, Khodja H, Fayard B, Flank A, Brisset F, Carriere M (2012). Accumulation, translocation and impact of TiO_2_ nanoparticles in wheat (*Triticum aestivum* spp.): influence of diameter and crystal phase. Sci Total Environ.

[CR14] Lee CW, Mahendra S, Zodrow K, Li D, Tsai Y, Braam J, Alvarez PJJ (2010). Developmental phytotoxicity of metal oxide nanoparticles to *Arabidopsis thaliana*. Environ Toxicol Chem.

[CR15] Lee W, Kim SW, Kwak JI, Nam S, Shin Y, An Y (2010). Research trends of ecotoxicity of nanoparticles in soil environment. J Toxicol Res.

[CR16] Lin D, Xing B (2007). Phytotoxicity of nanoparticles: inhibition of seed germination and root growth. Environ Pollut.

[CR17] Lin D, Xing B (2008). Root uptake and phytotoxicity of ZnO nanoparticles. Environ Sci Technol.

[CR18] Lv J, Zhang S, Luo L, Han W, Zhang J, Yang K, Christe P (2012). Dissolution and microstructural transformation of ZnO nanoparticles under influence of phosphate. Environ Sci Technol.

[CR19] Lyon DY, Adams LK, Falkner JC, Alvarez PJJ (2006). Antibacterial activity of fullerene water suspensions: effects of preparation method and particle size. Environ Sci Technol.

[CR20] Manzo S, Rocco A, Carotenuto R, De Luca PF, Miglietta LM, Rametta G, Di Francia G (2011). Investigation of ZnO nanoparticles' ecotoxicological effects towards different soil organisms. Environ Sci Pollut Res.

[CR21] Misra SK, Dybowska A, Berhanu D, Croteau MN, Luoma SN, Boccaccini AR, Valsami-Jones E (2012). Isotopically modified nanoparticles for enhanced detection in bioaccumulation studies. Environ Sci Technol.

[CR22] Misra SK, Dybowska A, Berhanu D, Luoma SN, Valsami-Jones E (2012). The complexity of nanoparticle dissolution and its importance in nanotoxicological studies. Sci Total Environ.

[CR23] Musante C, White JC (2010) Toxicity of silver and copper to Cucurbita pepo: differential effects of nano and bulk-size particles. Environ Toxicol 27:510–517 10.1002/tox.20667 22887766

[CR24] Nowack B, Ranville JF, Diamond S, Gallego-Urea JA, Metcalfe C, Rose J, Horne N, Koelmans AA, Klaine SJ (2012). Potential scenarios for nanomaterial release and subsequent alternation in the environment. Environ Toxicol Chem.

[CR25] OECD (1984) Guideline for testing of chemicals 208. Terrestrial Plants, Growth Test

[CR26] Pan B, Xing B (2010). Manufactured nanoparticles and their sorption of organic chemicals. Adv Agron.

[CR27] Persoone G, Wadiha K, Moser H, Rombke J (2010). Comparison between toxkit microbiotests and standard tests. Ecotoxicological characterization of waste: results and experiences of an international ring test.

[CR28] Piccinno F, Gottschalk F, Seeger S, Nowack B (2012). Industrial production quantities and uses of ten engineered nanomaterials in Europe and the world. J Nanoparticle Res.

[CR29] Pokhrel LR, Dubey B (2013). Evaluation of developmental responses of two crop plants exposed to silver and zinc oxide nanoparticles. Sci Total Environ.

[CR30] Rao KV, Sunandana CS (2008). Effect of fuel to oxidizer ratio on the structure, micro structure and EPR of combustion synthesized NiO nanoparticles. J Nanosci Nanotechnol.

[CR31] Schmidt J, Vogelsberger W (2006). Dissolution kinetics of titanium dioxide nanoparticles: the observation of an unusual kinetic size effect. J Phys Chem.

[CR32] Song U, Shin M, Lee G, Roh J, Kim Y, Lee EJ (2013). Functional analysis of TiO_2_ nanoparticle toxicity in three plant species. Biol Trace Elem Res.

[CR33] Stampoulis D, Sinha SK, White JC (2009) Assayphytotoxicity of nanoparticles to plants. Environ Sci Technol 43:9473–9479 10.1021/es901695c 19924897

[CR34] Tourinho PS, van Gestel CAM, Lofts S, Svendsen C, Soares AMVM, Looureiro S (2012). Metal-based nanoparticles in soil: fate, behavior, and effects on soil invertebrates. Environ Toxicol Chem.

[CR35] Van der Vliet L, Velicogna J, Princz J, Scroggins R (2012) Phytotoxkit: a critical look at a rapid assessment tool. Environ Toxicol Chem 31:316–323 10.1002/etc.743 22095428

[CR36] Von der Kammer F, Ferguson PL, Holden PA, Masion A, Rogers KR, Klaine SJ, Koelmans AA, Horne N, Unrine JM (2012). Analysis of engineered nanomaterials in complex matrices (environment and biota): general considerations and conceptual case studies. Environ Toxicol Chem.

[CR37] Waalewijn-Kool PL, Ortiz MD, van Gestel CAM (2012). Effect of different spiking procedures on the distribution and toxicity of ZnO nanoparticles in soil. Ecotoxicology.

[CR38] Walter I, Martınez F, Cala V (2006). Heavy metal speciation and phytotoxic effects of three representative sewage sludges for agricultural uses. Environ Pollut.

[CR39] Wu SG, Huang L, Head J, Chen D, Kong L, Tang YJ (2012). Phytotoxicity of metal oxide nanoparticles is related to both dissolved metals ions and adsorption of particles on seed surfaces. J Pet Environ Biotechnol.

[CR40] Zheng L, Hong F, Lu S, Liu C (2005). Effect of nano-TiO_2_ on strength of naturally aged seeds and growth spinach. Biol Trace Elem Res.

