# Supplementary material for: Phytotoxicity of nanoparticles—problems with bioassay choosing and sample preparation
Source: Environ Sci Pollut Res Int. 2014 Apr 23;21(17):10215–24. doi: 10.1007/s11356-014-2865-0 (PMC4138438; doi:10.1007/s11356-014-2865-0)
Supplement: Supplementary file 1 — (DOCX 1085 kb) [file 11356_2014_2865_MOESM1_ESM.docx]

Supporting information:

**SURFACTANTS SIGNIFICANTLY DECREASE THE TOXICITY OF ZnO, TIO_2_ AND Ni NANOPARTICLES TO *DAPHNIA MAGNA***

Patryk Oleszczuk^1*^, Izabela Jośko^1^

^1^Department of Environmental Chemistry, University of Maria Skłodowska-Curie, pl. M. Curie-Sklodowskiej 3, 20-031 Lublin, Poland

**Corresponding author: patryk.oleszczuk@poczta.umcs.lublin.pl*

Journal: Environmental Science and Pollution Research

Number of pages: 3 (including this page)

Number of figures: 4


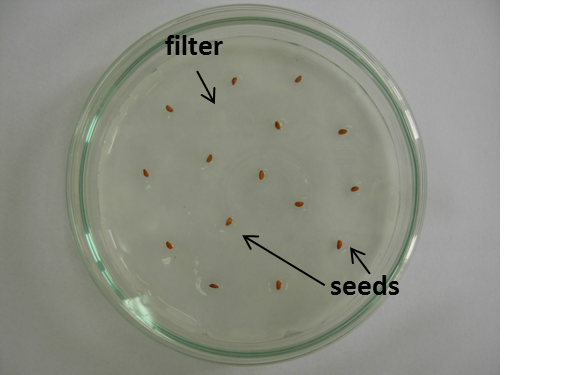


**Figure S1.** The germination/elongation test


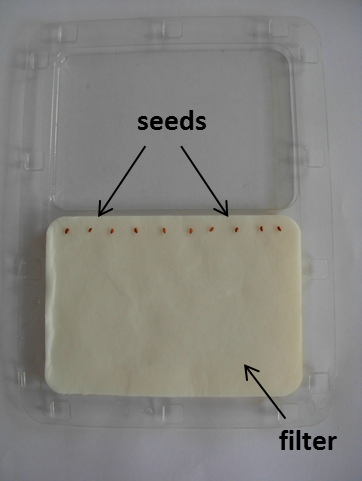


**Figure S2.** Phytotestkit F^TM^


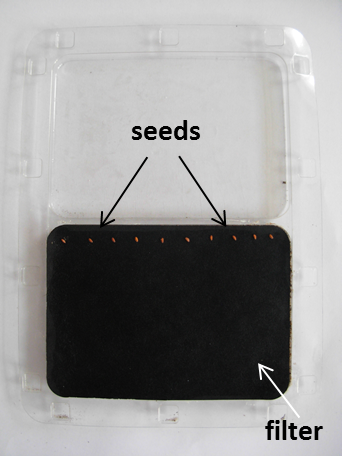


**Figure S3.** Phytotoxkit F^TM^


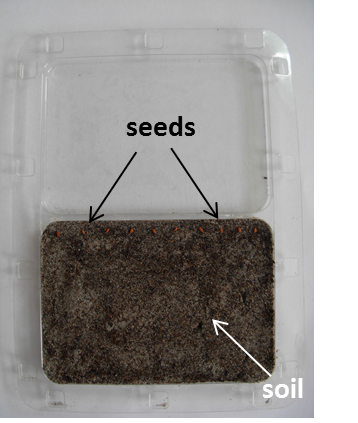


S4. Modified Phytotoxkit F^TM^
